# Size-dependent heavy metal and parasite bioaccumulation in *Mugil cephalus* from lake manzala: human health risks and fish histopathological lesions

**DOI:** 10.1038/s41598-025-19372-1

**Published:** 2025-09-23

**Authors:** Sally M. Salaah, Hanaa M. M. El-Khayat, Hanan S. Gaber

**Affiliations:** 1 National Institute of Oceanography and Fisheries, NIOF, Cairo, Egypt; 2 Environmental Research Department, Theodor Bilharz Research Institute, Giza, Egypt

**Keywords:** Mullet, *Myxobolus spp*, Ecotoxicology, Bioaccumulation, Tissue lesions, Carcinogenic risks, Freshwater ecology, Environmental sciences, Limnology

## Abstract

Pollutants, such as heavy metals (HM) and parasites, pose significant threats to aquatic environments. These contaminants can gather in fish, adversely affecting their health and potentially posing health risks to human consumers. Understanding the dynamics of these pollutants and their accumulative impact on fish of different sizes and the associated human health is crucial for the sustainability of aquatic ecosystems and food security. This study investigated the bioaccumulation of HM in the muscles of three different sizes of Mugil *M. cephalus* from the northeastern part of Lake Manzala, revealing significant size-related variations. Small-sized fish showed the highest levels of iron (Fe) and zinc (Zn), followed by medium-sized fish, while large-sized fish exhibited the lowest Fe and Zn concentrations. Conversely, larger fish accumulated higher levels of copper (Cu) and cadmium (Cd) compared to medium- and small-sized fish. Across all size groups of M. cephalus, the levels of both Fe and Cu exceeded the guidelines established by the Egyptian Organization for Standardization (EOS, 2005). The Metal Pollution Index (MPI) indicated that small-sized fish accumulate more HM, with medium- and large-sized fish showing lower MPI values compared to small-sized fish. In small- and medium-sized fish, Fe recorded the highest EDI values, followed by Zn > Cu > Pb > and Cd. In contrast, large-sized fish exhibited an EDI pattern of Fe > Cu > Zn > Pb > Cd for both normal and habitual consumers. Pearson correlation coefficients indicated strong negative correlations for Fe and Zn with fish size and strong positive correlations for Cd and Cu, while Pb showed a weak positive correlation. Principal Component Analysis (PCA) identified Cd, Cu, and Zn as primary contributors, with Fe and Zn showing negative loadings associated with smaller fish, and Pb had a significant positive loading in larger fish. The Target Hazard Quotient (THQ) and Hazard Index (HI) values revealed a size-related variation in health risks. Small-sized fish posed the highest non-carcinogenic risk for normal consumers; habitual consumers revealed significant health hazards (HI > 1) across all fish sizes, but it is particularly pronounced in consumers of small-sized fish. Although normal consumers recorded negligible carcinogenic risk, habitual consumers recorded low risk. The histological investigation showed significant alterations in the gills, liver, and kidneys of *M. Cephalus* is related to *Myxobolus* infection and HM accumulation, particularly Cd and Cu. Medium- and large-sized fish displayed more severe tissue alterations associated with higher HM load and increased prevalence of *Myxobolus* parasites. The findings emphasize that fish size is a critical factor affecting the synergistic interactions among heavy metal load and associated human health risks, parasitic infection, and histopathological lesions in fish. Underscoring the importance of continuous monitoring and risk assessment of HM and parasitic infections in aquatic ecosystems.

## Introduction

Heavy metals (HMs) are recognized as pervasive pollutants in various aquatic environments. Their prevalence is largely attributed to numerous anthropogenic activities, including industrial processes, agricultural practices, and urban development^1^. In aquatic environment, fish accumulate pollutants that exist in the surrounding environment by several time in their tissue^2^. At safe levels, essential HM including iron (Fe), zinc (Zn), and copper (Cu) are needed for fish survival and growth^3^. However, the biological contribution of other HM such as Pb and Cd is still unidentified. HM are mostly absorbed through adjacent water, sediments, and food and accumulated in fish tissue^4^.

Consuming HM-polluted fish can cause severe health problems. Lead (Pb) exposure can damage the brain and kidneys, causing learning difficulties and behavioral issues in children^5^, while cadmium (Cd) is associated with kidney dysfunction, bone weakening, and cancer risks^6^. Chronic ingesting of contaminated fish leads to bioaccumulation in the human body, increasing the risk of skin lesions, organ damage, and carcinogenesis and immune suppression^7^.

Fish accumulate HM based on the concentration of metals, duration of exposure, habitat, feeding habits, and characteristics of the surrounding water and sediment^8^. Moreover, some biological requirements and properties of fish such as size, which have been recognized to affect the HM accumulation rate^9^. Consequently, advanced understanding on the associations between the biological and biometric properties of fish and HM bioaccumulation load could be beneficial from the ecological and human health perspective.

Manzala Lake contributes over 30% of Egypt’s commercial fish supply. However, in the past six decades, the lake has experienced significant pollution from various sources, including agriculture, sewage, and industrial wastewater, particularly along its southern and western borders^10^. These contaminants have severely polluted the lake, leading to a substantial decline in commercial fish production and the degradation of fish health and natural resources^11,12^.

Lake Manzala receives drainage water from six major heavily polluted drains: Bahr El-Baqar, Hadous, El-Serw, Ramsis, Mataria, and Faraskur, with a combined total discharge of approximately 4000 million m^3^ per year^13^. The Bahr El-Baqar and Hadous drains contribute approximately 75% of the total waste input into Lake Manzala, with pollution sources including both treated and raw industrial wastewater, sewage, and agricultural drainage^14^. Additionally, the Hadous, El-Serw, Ramsis, and Faraskur drains primarily discharge agricultural water into the lake, while the Mataria drain is responsible for discharging sewage waste^15^.

Mugilids, belonging to the Teleostei family *Mugilidae*, are a diverse group of fish known for their high opportunism and their presence in a wide range of habitats worldwide. They exhibit a remarkable ability to tolerate varying abiotic conditions, including salinity, temperature, sedimentary regimes, turbidity, and dissolved oxygen levels^16^. Consequently, mullets are commonly found in coastal areas, estuaries, lakes, and lagoons, spanning both tropical and temperate regions.

The adaptability and ubiquity of mullets contribute to their exceptional survival skills and significant ecological roles. They serve as vital participants in the transfer of matter and energy between trophic levels, playing a key role in ecosystem functioning. Mullets are involved in nutrient cycling and energy flow due to their consumption of detritus, algae, plankton, and benthic organisms^17^.

Guimarães^18^ stated that the wide distribution and significant ecological role of mullets make them susceptible to parasitic infections and diseases, as they are susceptible for various pathogens, including parasites, bacteria, and viruses. Moreover, stressful conditions such as overpopulation, poor water quality, and pollutants further increase their vulnerability.

Mullet farming in Egypt predominantly depends on the collection of wild seeds. Initially, the government set up seed collection stations to provide fingerlings to licensed fish farms. Over time, this activity has transitioned into a private enterprise with minimal government involvement^19^. The three most commonly farmed mullet species in Egypt are grey mullet (*M. cephalus*), thin-lipped grey mullet (Liza ramada), and dotted grey mullet (*V. seheli*) are preferred due to their fast growth rates and high market demand. The striped mullet (*M. cephalus*) holds significant importance as a key fish species and serves as a primary cash crop for traditional artisanal fisheries scattered across various regions of Egypt^20^.

Myxozoans are parasitic cnidarians that extensively spread in aquatic ecosystems, using fish as a temporary vertebrate host^21^. Given the ecological and economic threats posed by many species in this group to wild and reared fish populations, studying myxozoan biodiversity in mullets is essential for sustaining natural stocks and enhancing aquaculture production^22^.

Infected mullets with myxobolid parasites do not experience any noteworthy adverse effects other than the presence of the parasites, without instances of mortality. However, in the context of fish farms, an infection by these parasites could pose a potential risk, although the specific risks are not specified in the available information^23^.

The life cycle of myxobolid parasites involves migrating through the fish’s vasculature and establishing a persistent coelozoic sporogonic phase within the renal tubules of the posterior kidney. Tolerant hosts excrete mature malacospores into the environment, which can infect bryozoans and complete the life cycle^24^. Surviving fish can develop a certain level of protective immunity and may be able to clear the infection and regenerate damaged tissues^25^.

Ecoxicological research have mostly focused on the HM load of raw muscle in fish species; however, the changes of such toxic components and the related health hazards have yet to be thoroughly addressed^26^. Continuous assessment of HM and pathogens such as myxobolid parasites in aquatic ecosystems is essential due to the significant ecological and health risks associated with these contaminants. Previous study reported that HM contamination can influence the prevalence and intensity of parasitic infections in fish^27^, suggesting that HM may impact the immune response of fish, making them more susceptible to parasitic infections. This fish-parasite-metals interactions have been anticipated as an effective monitoring technique for evaluating the health of the fish ecosystem, with parasites signifying the presence of numerous pollutants in aquatic habitats, including noxious metals and sewage contaminants^28^. Understanding this link is crucial for developing effective strategies to manage both HM pollution and parasitic infections in aquatic ecosystems.

This study was conducted to multifaceted an approach provides comprehensive insights into the environmental and health impacts of heavy metal contamination and parasitic infections on this crucial fish species. Through assessing the accumulation of heavy metals in various sizes of *M. cephalus* from the northeastern part of Lake Manzala. The primary objectives were to investigate the relationship between heavy metal accumulation and fish size and to evaluate the associated health risks of consuming these fish. Additionally, the study aimed to establish a link between Myxobolid parasite infections, fish size, heavy metal load, and histopathological lesions in the gills, liver, and kidney of *M. cephalus*.

## Materials and methods

### Study area

**Fig. 1:**
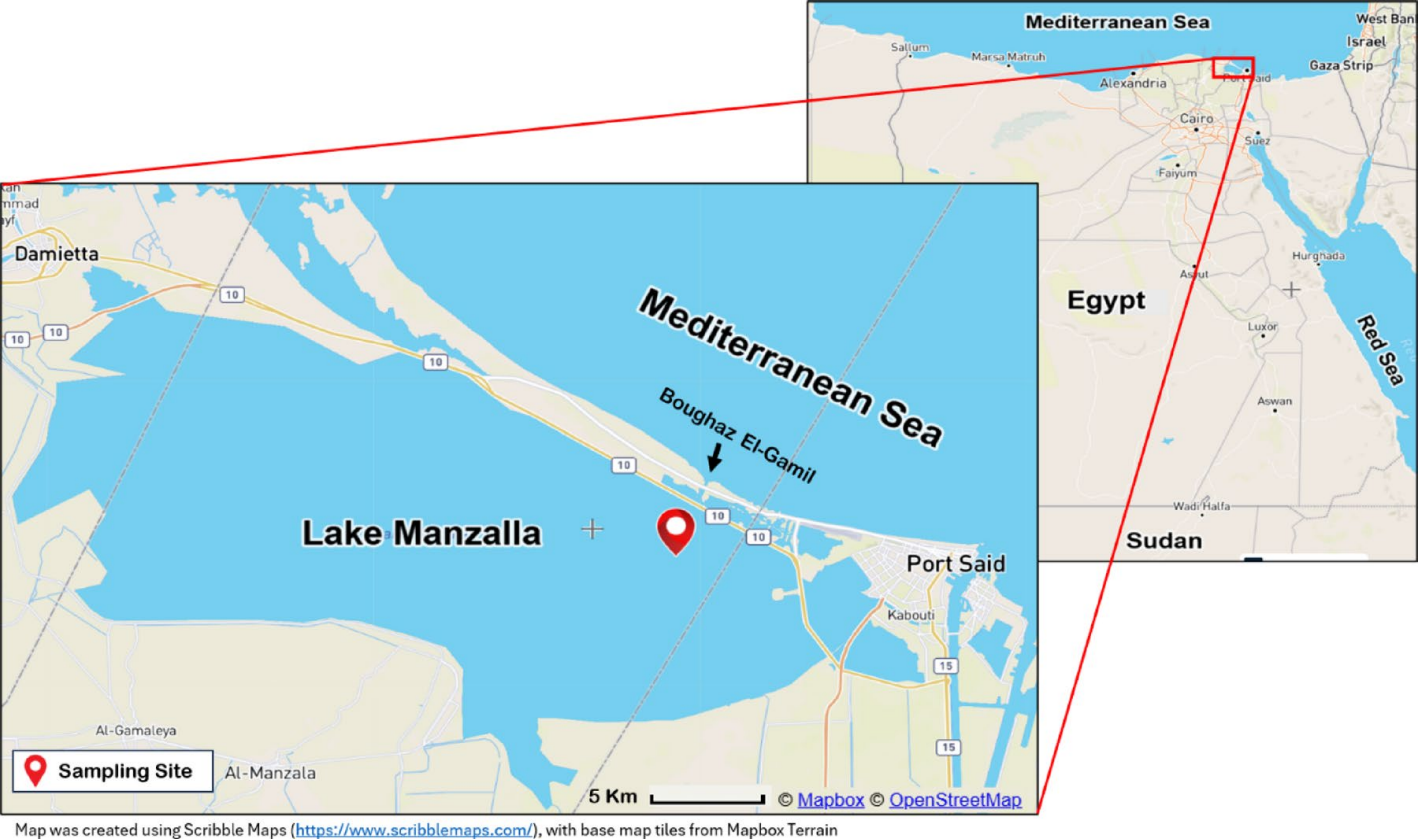
Manzala Lake map showing the study area.

Manzala Lake is a quadrilateral basin with average length of 60 km, width of 40 km, and depth of 1.3 m. It is situated between latitudes 31°10′, 31°40′N and longitudes 31°50′ and 32°25′E. The lake is detached from the Mediterranean Sea by a sandy beach rim, which interrupted by inlets known as boughaz, allowing the sea water to inter the lake at the northeast corner of the lake (Fig. 1), near pollution influx points (Bahr El-Baqar drain). The map demonstrating the sampling locations was generated using Scribble Maps (https://www.scribblemaps.com/), an online mapping tool, with geographic coordinates based on field data, using Mapbox Terrain base map. A total of thirty *M. cephalus* fish samples were assembled from the boughaz area, the northeastern inlet of Lake Manzala, near Port-Said Government Area, Egypt. fish were collected in August 2023. The fish samples were sorted equally into three size categories (small, medium, large) as shown in Table 1.

**Table 1:** Average weight and length (mean ± SD) for each size class of *M. cephalus* from Manzala lake.

	Small	Medium	Large
Length (cm)	12.5 ± 1.4	17.5 ± 1.9	22.5 ± 2.1
Weight (g)	33.18 ± 3.5	75.64 ± 4.1	138.4 ± 4.7

### Determination of HM bioaccumulation in *M. cephalus*

The edible tissues (muscles) were dissected from fish samples represented each category to appraise the heavy metals (HM) contents. The muscle samples were dried and subjected to digestion using the method described by APHA^29^. Where one gram of the dried powder was digested in acidic solution (1:1 nitric and perchloric acid). The obtained mixture was heated on a hot plate at 80–90 °C until the sample is clear. After cooling, the solution was filtered and de-ionized water used to obtain desired final volume. The resulting digests were stored in plastic bottles till analysis. Iron (Fe), zinc (Zn), copper (Cu), cadmium (Cd), and lead (Pb) analysis were conducted using an Inductively Coupled Plasma Emission Spectrometer (ICAP-6300 Duo), according to the guiding principle of APHA^30^. HM concentrations in muscles were expressed as mg/Kg of dry weight.

### Assurance/quality control (QA/QC)

To ensure and regulate quality, duplicate prepared samples, reagent blanks, and confirmed reference material were used at random throughout the measurement process for each digestion batch. The observed high sample recovery between 95 and 101%^31^.

### Metal pollution index (MPI)

MPI was conducted following the equation of Usero^32^ in the muscle tissues from *M. cephalus*.

1 $${\text{MPI}} = \left( {Cm_{{Fe}} + Cm_{{Mn}} + Cm_{ \ldots } } \right)^{{1/n}}$$

where Cm is the concentration of specific HM (mg/Kg).

### Health risk assessment

#### Estimated daily intake (EDI) of HM

The EDI is calculated relative to the rate of fish consumption for adults over lifespan as stated by Bo^33^.

2 $${\text{EDI~}} = \frac{{{\text{Mc}} \times {\text{IR}}}}{{{\text{BW}}}}$$

where Mc is metal concentration; IR = 0.05 kg/day (normal) and 0.15 kg/day (habitual consumers), followed^2,34^.

#### Non carcinogenic risk

##### Target hazard quotient (THQ)

The THQ is typically used to appraise the non-carcinogenic risk of ingested metals refer to USEPA^34,35^.

3 $${\text{THQ~}} = \frac{{{\text{EDI}} \times {\text{EF~}} \times {\text{ED}}}}{{{\text{R}}f{\text{D}} \times {\text{BW}} \times {\text{AT}}n}}$$

where RfD is the reference oral dose of metal intake (mg/kg day); Atn is the averaging time for non-carcinogens exposure according to USEPA^35^.

##### Hazard index (HI)

The HI is used to estimate the total hazard persuaded by the metal’s exposure for.

4 $${\text{HI}} = \mathop \sum \limits_{{i = 1}}^{n} THQ_{{Fe}} + THQ_{{Mn}} + THQ_{{Zn}} + \cdots$$

#### Carcinogenic risk

##### Target cancer risk (TR)

TR is used to analyze the potential carcinogenic risk linked to the intake of certain elements (Cd, Pb and Ni) from fish.

5 $${\text{TR}} = \frac{{{\text{EDI}} \times CPS_{0} \times {\text{EF}} \times {\text{ED}}}}{{{\text{AT}}c}}$$

where ATc is the averaging time for carcinogens exposure according to USEPA^35^. TR and ∑TR are considered tolerable at ≤ 1.0 × 10^−6^ and 1.0 × 10–5^35^.

### Histologic analysis

Fish mullet (*M. cephalus)* samples were collected with the help of professional local fishermen. Fish were dissected in the field and the vital organs (gill, liver, and kidney) were dissected and equipped for the histological analysis. Tissue samples were fixed 24 h using 10% formalin solution, embedded in paraffin, and sectioned into thin slices (~ 4–6 μm) mounted on glass slides. The obtained slides were submerged in staining hematoxylin solution for 10 min, followed by eosin staining for 3 min followed the method of Allen (1992). A light microscope was used to investigate the samples.

### Statistical analyses

The data of HM in muscles of *M. cephalus* were articulated in mean ± standard deviation (mean ± S.D) of thirty fish (*n* = 10 for each group). one-way analysis of variance was used to explore data statistically explored followed by Tukey’s test. If *P* ≤ 0.05, the difference was considered significant. To evaluate the relationship between fish size (small, medium, and large) and HM concentrations, the Pearson correlation coefficient (r) was computed. principal component analysis (PCA) was done to find the principal components (PCs) and the HM contributing to computing these PCs, using the statistiXL software (www.statistixl.com)^36^.

The relationship between the elements in fish species was examined through.

## Results

### HM bioaccumulation in *M. cephalus*

The bioaccumulation of HM in the muscles of *M. cephalus* exhibited a remarkable size-related variation, as shown in Table 1. Small-sized *M. cephalus* recorded the highest Fe and Zn content among all analyzed fish, followed by the medium-sized fish, while large-sized fish exhibited the lowest Fe and Zn concentrations. Larger *M. cephalus* fish accumulated higher levels of Cu and Cd in their edible tissues compared to medium- and small-sized fish.

The levels of both Fe and Cu exceeded the guidelines established by the Egyptian Organization for Standardization and Quality Control^37^ across all *M. cephalus* size groups, while Zn surpassed these guidelines in small- and medium-sized fish. In contrast, both Cd and Pb levels in *M. cephalus* muscles were within the limits detailed by EOSQC guidelines^37^ (Table 2). The MPI was calculated using Eq. 1, revealed that small-sized fish accumulate more HM within their edible tissues followed by medium- and large-sized fish, recording lower MPI by 52.91 and 63.34% compared to small-sized fish, respectively (Table 2).

**Table 2:** Heavy metals bioaccumulation (mg/Kg) and metal pollution index (MPI) in different sizes of *Mugil cephalus* from Manzala lake.

	Small	Medium	Large	Guidelines (mg/Kg)
Fe	190.38 ± 70.18^a^	71.33 ± 19.55^b^	55.65 ± 1.31^c^	30
Zn	30.18 ± 3.82^a^	26.88 ± 2.12^a^	11.30 ± 7.46^c^	20
Cu	10.70 ± 0.5^a^	9.50 ± 0.1^b^	18.55 ± 0.31^a^	0.5
Cd	0.03 ± 0.5^a^	0.04 ± 0.01^a^	0.05 ± 0.01^a^	0.05
Pb	0.87 ± 13.3^ab^	1.45 ± 1.41^a^	0.98 ± 4.81^a^	40
MPI	46.43	21.84	17.31	

Data were tabulated as mean ± S.D (*n* = 10 fish). PL: Permissible limits according to the standards of E.O.S.Q.C. (2005). Data within a row with similar superscripts have no significant difference between them (Tukey’s Test, *P* ≤ 0.05)

### EDI values of *M. cephalus* consumers

The EDI (Eq. 2) for normal consumers varied between 8.4E-02 and 1.34E-05 in small fish, 3.18E-02 and 1.87E-05 in medium-sized fish, and 2.48E-02 to 2.07E-05 in large-sized fish. Habitual consumers recorded higher EDI values, especially when consuming small-sized fish, with a range of 6.13E-05 to 3.86E-01. Medium-sized fish recorded intermediate EDI values, ranging from 1.45E-01 to 8.54E-05 (Table 3).

**Table 3:** Estimated daily intake (EDI) of HM from different sizes of *M. cephalus* from of Manzala lake.

		Small	Medium	Large
Fe	Normal	8.49E-02	3.18E-02	2.48E-02
	Habitual	3.87E-01	1.45E-01	1.13E-01
Zn	Normal	1.34E-02	1.20E-02	5.04E-03
	Habitual	6.14E-02	5.47E-02	2.30E-02
Cu	Normal	4.77E-03	4.23E-03	8.27E-03
	Habitual	2.18E-02	1.93E-02	3.77E-02
Cd	Normal	1.34E-05	1.87E-05	2.07E-05
	Habitual	6.10E-05	8.54E-05	9.46E-05
Pb	Normal	3.88E-04	6.48E-04	4.38E-04
	Habitual	1.77E-03	2.96E-03	2.00E-03

In small- and medium-sized fish, Fe recorded the highest EDI values, followed by Zn > Cu > Pb > and Cd. In contrast, large-sized fish exhibited an EDI pattern of Fe > Cu > Zn > Pb > Cd for both normal and habitual consumers (Table 3).

### The pearson correlation coefficients in *M. cephalus*

**Fig. 2:**
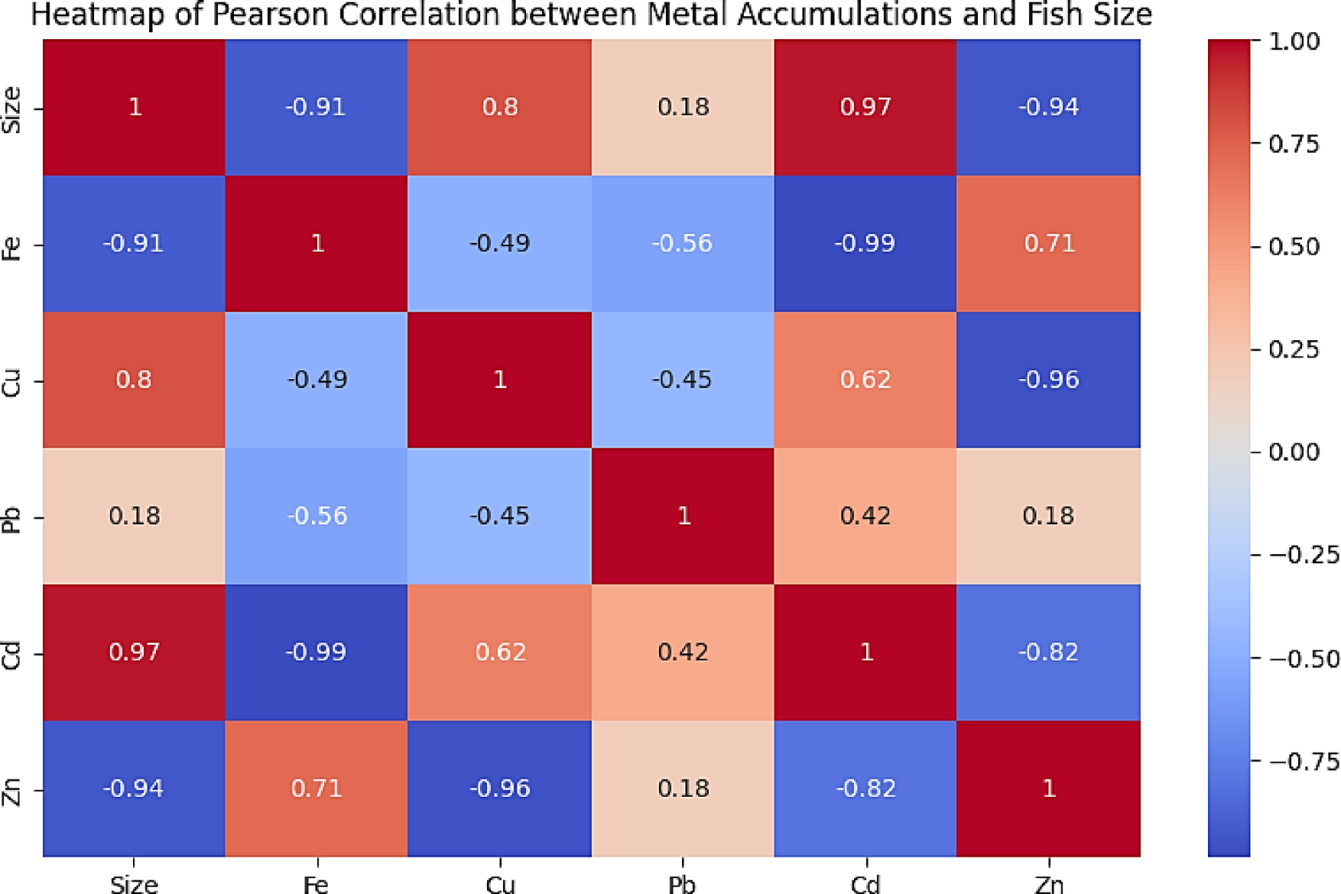
Heatmap showing Pearson correlation between HM accumulation in *M. cephalus* and fish size.

The Pearson correlation coefficients was used to understand the strength and direction of the relationship between HM accumulations in *M. cephalus* and fish size. Both Fe and Zn with fish size (*r* = -0.91 and − 0.94, respectively), indicating that smaller fish tend to have higher concentrations of these metals. Cd exhibited a strong positive correlation (*r* = 0.97) with fish size, signifying reliable and substantial increase in Cd related to fish size, that larger fish tend to have higher concentrations of Cd. Pb recorded a weak positive correlation (*r* = 0.18), suggesting a slight increase in Pb concentration with increasing fish size. Cu displayed a strong positive correlation (*r* = 0.80), indicating a reliable increase in Cu concentration with fish size. This means that as the fish grow larger, their tissues tend to accumulate more Cu (Fig. 2).

### Principal component analysis across different *M. cephalus* sizes

**Fig. 3:**
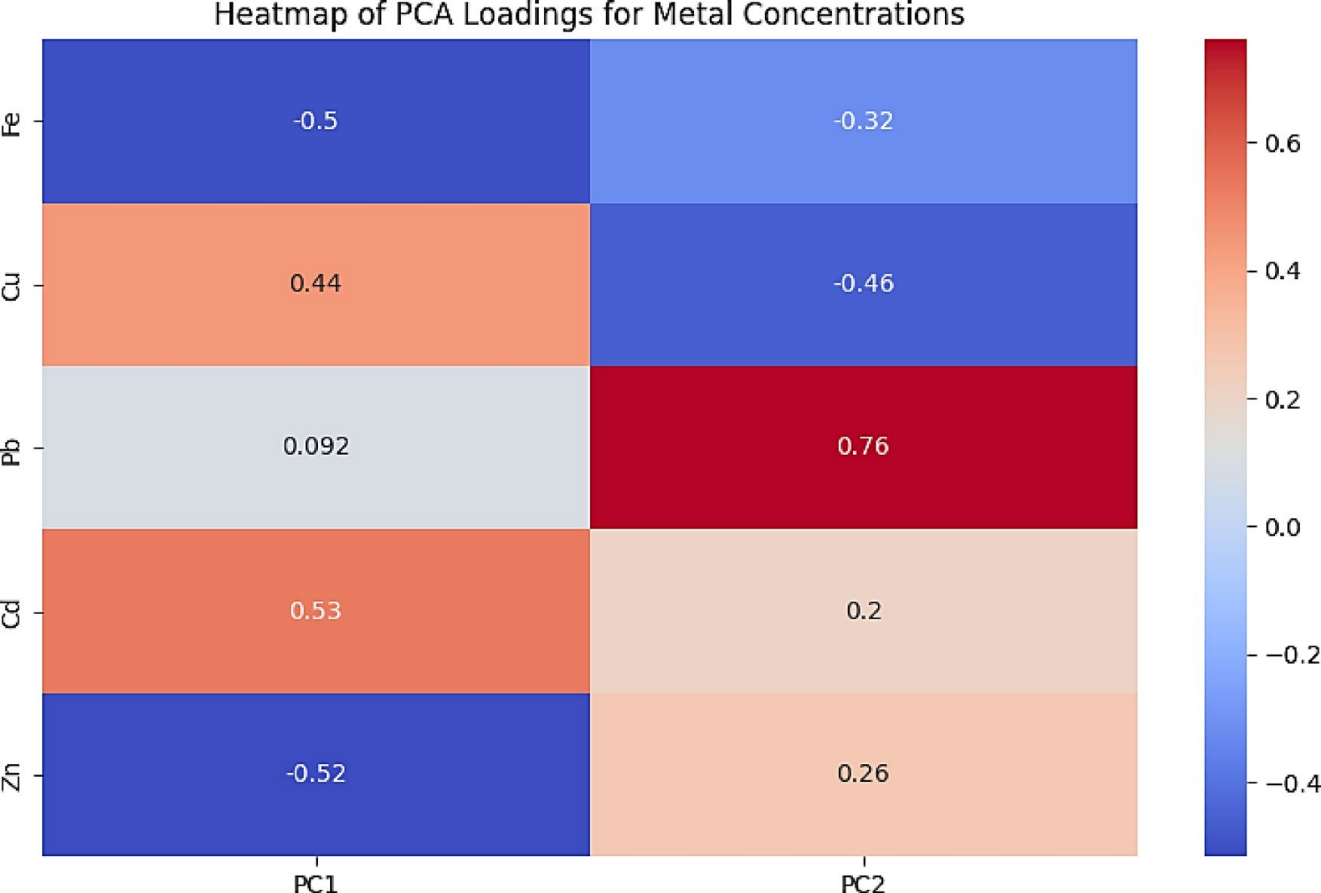
Heatmap showing Principal Component Analysis (PCA) loadings for HM accumulation in *M. cephalus* and fish size.

The Principal Component Analysis (PCA) was conducted to reveal the underlying patterns in HM concentrations across different fish sizes. PCA revealed that Cd, Cu, and Zn are the primary contributors (PC1). while Fe and Zn recorded negative loadings indicated that higher concentrations of these metals are dominated smaller fish. Giving the principal Component 2 (PC2), Pb is the most significant contributor. Positive loadings for Pb, Cd and Zn (0.76,0.2, and 0.26) suggested that higher concentrations of these metals are associated with larger fish sizes in this trend (Fig. 3).

**Fig. 4:**
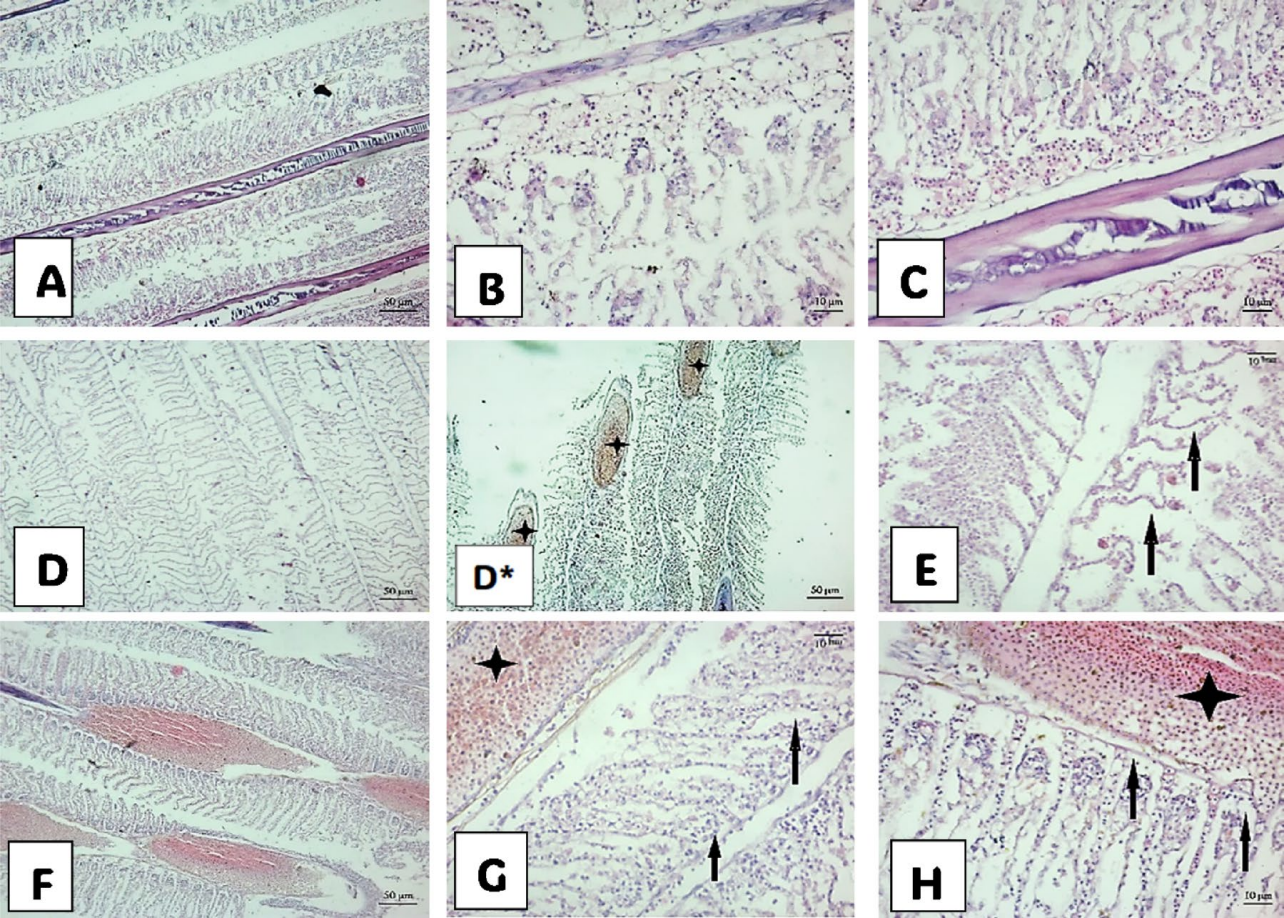
Photomicrograph of the gill histological structure of *M. cephalus* from Manzala Lake. (**A**), (**B**), and (**C**) show gills of small-sized fish; (**A**) Mucus secretion with lamellarFusion and necrotic change of of interlamellar epithelium. (**B**) Necrotic change of gill lamellae. (**C**) Necrotic change of gill lamellae, Destroyed pillar system. (**D**), (D*), and (**E**) represent gills of midium-sized fish; (**D**) Mucus laden and congestion of apical gill filament (star), fusion of gill lamellar in (D*). (**E**) Lamellar fusion, and necrosis of lamellar epithelial cells (arrows). **F**, (**G**), and (**H**) show gills of large-sized fish; (**F**) Dilation along gill filaments with severe congestion. (**G**) Inflammatory cells within the entire length of the gill filament with spores (star) and edema (arrow) fusion of lamellae. (**H**) Inflammatory cells with spores(star) and edema (arrows).

**Fig. 5:**
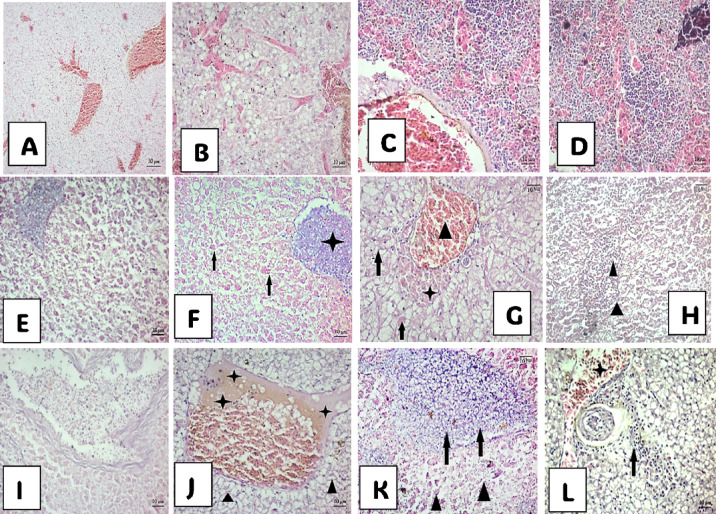
Photomicrograph of the liver histological structure of *M. cephalus*. (**A**)–(**D**) showing liver of small-sized fish; (**A**) Multifocal congestion between hepatocytes. (**B**) Severe congestion, fibrolysis and fatty necrotic change. (**C**) Dilated vein with congestion and congestion between hepatocytes. (**D**) Intracytoplasmic inclusion bodies and hemosiderin. (**E**)–(**H**) showing liver of medium-sized fish; (**E**) Multifocal granulomas and intracytoplasmic inclusion bodies. (**F**) Dilated vein filled with granulomas(star) and intracytoplasmic inclusion bodies (arrows). (**G**) Enlargement of bulk of undifferentiated cells, granulomas (arrows) excessive erythrocytes out of the vessel (head arrow). (**H**) Excessive erythrocytes out of the vessel (head arrow). (**I**)–(**L**) showing liver in large-sized fish; (**I**) Dilated vein with Erythrocyte infilteration. (**J**) Dilated vein with hyaline degeneration(stars) and hepatic vascular degeneration and pycnotic nuclei (head arrow). (**K**) Multifocal chronic hepatitis (arrows) and undifferentiated cells around vein (star) congestion dilated vein (head arrow). (**L**) Cyst around with connective tissues, intra vascular congestion and aggregation of spores between hepatocyte cells (arrow).

**Fig. 6:**
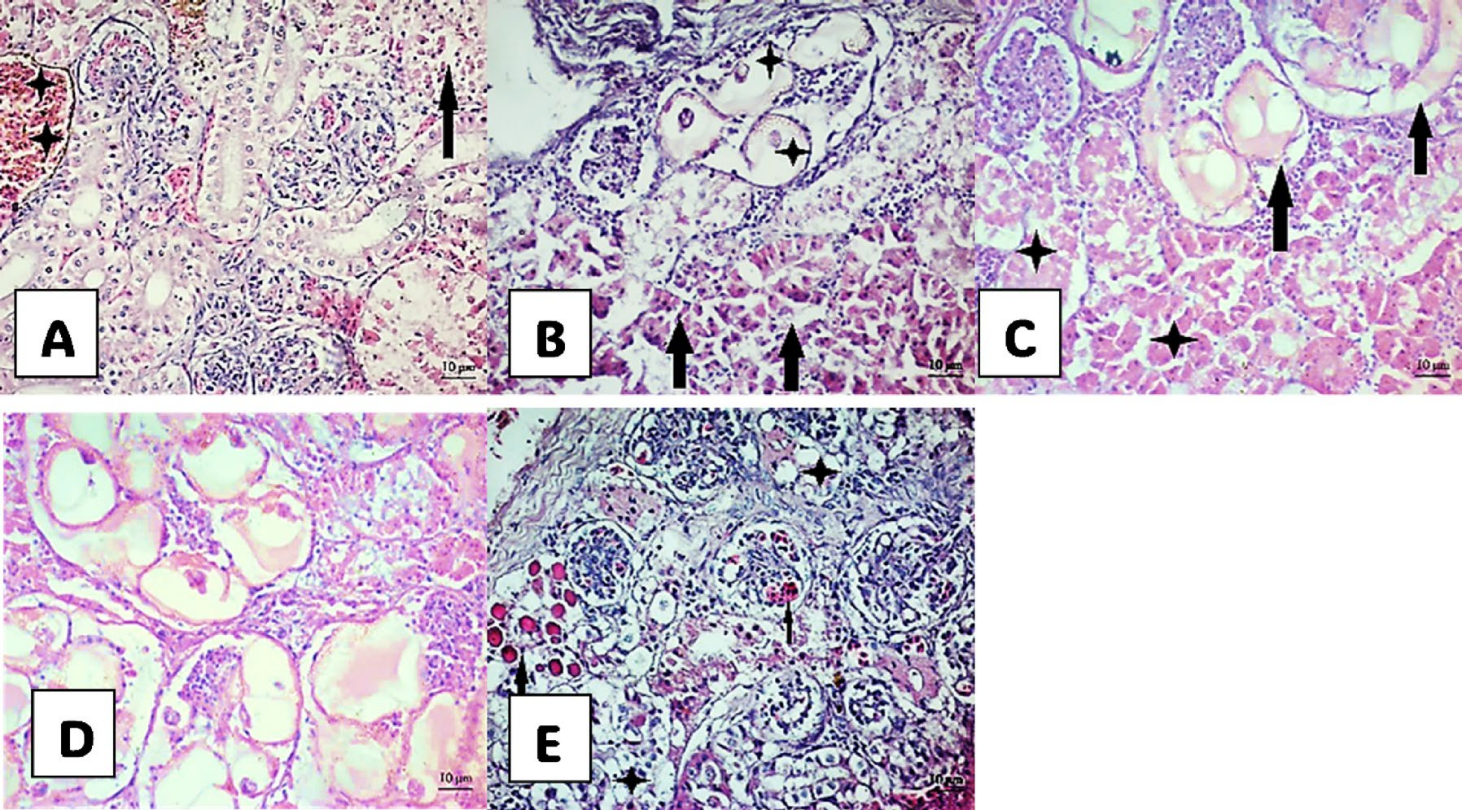
Photomicrograph of kidney histological structure of *M. cephalus*. (**A**) Congested dilated vein (star). Blood cells and spores in bowman’s capsule (arrow) in small-sized fish. (**B**)–(**D**) show kidney of medium-sized fish; (**B**) Fibrous plasmodia-like structure in the kidney interstitium with the residue of *Myxobolus spp*. degenerated developmental stages and spores, and plasmodium in glomeruli (star). necrotic renal tubules (arrows). (**C**) Glomeruli parasitized by *Myxobolus spp* (arrows) and degenerated renal tubules(stars). (**D**) Cysts with numerus plasmodmium of parasite. (**E**) Spores distributed in the kidney interstitium (stars)and necrotic tubules with hyaline around capsules and foreign cells, intra cytoplasmic inclusion bodies (arrows) in large-sized fish.

Fe displayed a moderate negative loading on PC1(-0.50), indicating that higher Fe concentrations are associated with smaller fish sizes. Fe displayed a moderate negative loading on PC2 (-0.32), suggesting that as the size of the fish changes, Fe concentration varies inversely with both principal components.

Cu recorded a moderate positive loading on PC1 (0.44), indicating that higher Cu concentrations are associated with larger fish sizes. Cu displayed a moderate negative (-0.46) loading on PC2, suggesting that Cu influenced the secondary trend in an opposite direction compared to its influence on PC1. Pb revealed a low positive loading on PC1 (0.09) and a high positive loading (0.76) on PC2, suggesting that higher lead concentrations are associated with larger fish size.

Cd showed a high positive loading (0.53) on PC1, indicating that higher Cd concentrations are associated with larger fish sizes. Cd displayed a low positive loading PC2 (0.20) on PC2, suggesting a modest influence on the secondary trend. Zn revealed a high negative loading (-0.52) on PC1, signifying that higher Zn concentrations are associated with smaller fish sizes. Zn has a moderate positive loading (0.26) on PC2, suggesting that higher Zn concentrations linked to larger fish sizes in this trend (Fig. 3).

### THQ values of *M. cephalus consumers*

The THQ values for normal consumer ranged from 1.29E-01 to 1.34E-02, 2.16E-01 to 1.87E-02, and from 2.07E-01 to 3.54E-02 in small-, medium-, and large-sized fish, respectively. The habitual consumers’ THQ values varied between 5.91E-01 and 6.10E-02, 9.86E-01 and 1.82E-01, 9.43E-01 and 9.46E-02 for small-, medium-, and large-sized fish, correspondingly (see Table 4).

**Table 4:** Hazard quotient (THQ) and hazard index (HI) of HM from different sizes of *M. cephalus* from Manzala lake.

THQ				
Metal	consumption rate	Small	Medium	Large
Fe	Normal	1.21E-01	4.54E-02	3.54E-02
	Habitual	5.53E-01	2.07E-01	1.62E-01
Zn	Normal	4.48E-02	3.99E-02	1.68E-02
	Habitual	2.05E-01	1.82E-01	7.66E-02
Cu	Normal	1.19E-01	1.06E-01	2.07E-01
	Habitual	5.44E-01	4.83E-01	9.43E-01
Cd	Normal	1.34E-02	1.87E-02	2.07E-02
	Habitual	6.10E-02	8.54E-02	9.46E-02
Pb	Normal	1.29E-01	2.16E-01	1.46E-01
	Habitual	5.91E-01	9.86E-01	6.66E-01

HI				
consumption rate	Small	Medium	Large	
Normal	0.52	0.46	0.42	
Habitual	1.97*	1.94*	1.92*	

*HI ≤ 1.0.

###  HI values of *M. cephalus consumers*

The HI values (Eq. 4) recorded a size-related variation, where small-sized fish recorded the highest risk for normal consumers followed by medium and large-sized fish, as demonstrated in Table 3. The HI indicated a non-carcinogenic health risk (HI ≥ 1.0) for habitual consumers of *M. cephalus* fish from boughaz area, regardless the size of fish. Small-sized fish recorded highest HI values followed by medium- and large-sized fish for habitual consumers.

### TR values of *M. cephalus consumers*

The TR values (Eq. 5) for both Cd and Pb revealed negligible carcinogenic risks (TR ≤ 10^−6^) for normal consumers of *M. cephalus* from boughaz area, nevertheless the size of fish. While habitual consumers Habitual consumers faced non-carcinogenic risks (HI > 1) across all sizes, recording a low carcinogenic risk (TR ≤ 10^−5^) for Cd and Pb, as revealed in Table 5. The total TR demonstrated a low carcinogenic risk (∑TR ≤ 10^−5^) for both normal and habitual consumers across all *M. cephalus* size groups, except for normal consumers of small-sized fish, revealing a negligible cancer risk (∑TR ≤ 10^−6^), as shown in Table 5.

**Table 5:** The target cancer risk (TR) and total TR (ΣTR) of HM of different sizes of *M. cephalus* from Manzala lake.

TR		Small	Medium	Large
Cd	Normal	5.08E-06	7.11E-06	7.88E-06
	Habitual	2.32E-05	3.25E-05	3.59E-05
Pb	Normal	3.30E-06	5.51E-06	3.72E-06
	Habitual	1.51E-05	2.52E-05	1.70E-05

∑TR				
Normal	8.38E-06	1.26E-05	1.16E-05	
Habitual	3.83E-05	5.76E-05	5.29E-05	

Cancer risk considered negligible at ≤ 10^−6^: low at ≤ 10^−5^: medium at ≤ 10^−4^: high at ≤ 10^−3^ and very high ≥ 10^−3^.

### Histological investigation

The present histological study focused on the alterations in the gills, liver, and kidneys of *M. cephalus* fish of three different sizes obtained from the northern east region of Lake Manzala. These alterations were analyzed in relation to Myxobolus infection and the accumulation of HM (Fe, Zn, Cu, Cd, and Pb) in their muscles.

fish displayed various histopathological alterations in all organs in according to fish size, taking into account the degree of immunity of the fish against the parasite.

#### The histopathological alteration in the gills of *M. cephalus*

The gills of *M. cephalus* fish displayed various histopathological alterations, as shown in Fig. 4. These changes included hyperplasia, mucus secretion with lamellar fusion, necrotic modifications in the interlamellar epithelium, severe congestion, and the presence of spores across the gill filament. Blood vessels appeared dilated, with highly compressed endothelial cells. Additionally, edema was noticeable at the base of the gill lamellae, with fluid infiltration into the tissues.

#### The histopathological alteration in the liver of *M. cephalus*

The liver samples of the studied fish, presented in Fig. 5, showed red blood spots, excessive erythrocytes outside the vessel, enlargement of cysts with granuloma development from connective tissue or fibrotic capsules, severe congestion, dilated veins, and multifocal chronic hepatitis. Focal areas of lymphocytic aggregation migrated from dilated blood vessels toward necrotic hepatic cells.

### The histopathological alteration in the kidney of *M. cephalus*

The histopathological features of the kidneys, shown in Fig. 6, included congested dilated veins, necrotic tubules with hyaline around Bowman’s capsule, spores, intracellular inclusion bodies, and numerous cysts filled with plasmodia distributed in the kidney interstitium and glomeruli parasitized by *Myxobolus spp*.

## Discussion

One of the significant goals in the United Nations Agenda 2030 is food security, which is a multi-dimensional goal for Sustainable Development goals (SDGs); since its related to many SDGs such as zero hunger (2nd goal) and good health and wellbeing (3rd goal)^38^. Food ingestion is the typical pathway of exposure to many health risks in humans, such as metal exposure. In low- and middle-income nations, malnutrition is a substantial challenge.

Fish play a significant role in enhancing food and nutrition security through addressing these global challenges. Since fish is a rich source of many fundamental nutrients for human including high-quality proteins, omega-3 fatty acids, minerals, and vitamins^39^. Beyond its nutritional benefits, fish contribute in a significant way to food availability by providing a sustainable and affordable source of animal protein, especially in low-income countries. In Egypt, fish is an essential constituent in the Egyptians’ regime owing to its availability and affordability.

This study was conducted to establish a relationship between tissue HM concentrations of *M. cephalus* and fish size. The present study revealed a size-specific patterns of HM accumulation in *M. cephalus* from Manzala Lake. The results indicate that most statistically significant cases demonstrate strong relationships between fish sizes and HM load in *M. cephalus* muscles. These findings are in agreement with a previous study by Al-Yousuf et al.^40^ who stated a positive correlation between the bioaccumulations of Cu and Cd in fish and both fish length and weight.

### HM accumulation in *M. cephalus*

HM accumulation in the organs of aquatic animals can also be affected by environmental supplies, size, sex, and maturation processes^26^. Additionally, Widianarko et al.^41^ investigated the relationship between HM concentrations and the size of fish, reporting a substantial decline in Pb concentrations with increasing size, while Cu and Zn concentrations persisted unaffected by body weight. Other studies have also identified negative relationships between fish size and HM concentrations^42^.

Despite these outcomes, there is no definitive or established relationship between HM concentration and fish size. It has been observed that HM accumulation in fish reaches a steady state after a certain age^43^. This steady state suggests that concentrations of Cu and Zn essential for fish metabolism are regulated and maintained at specific levels. However, the dilution of tissue HM concentrations typically associated with growth and lower metabolic activity in older individuals^26^ may not be evident if environmental HM concentrations exceed the regulatory capacity of these individuals.

Furthermore, older fish possess more advanced enzymatic systems and enhanced elimination pathways such as excretion or reproduction compared to younger fish. It is also important to note that changes in diet as fish grow larger can contribute to decreased contaminant concentrations since the accumulation of contaminants is influenced by the dietary formula^44^. Consequently, adult fish that consume prey with lower HM contamination levels will be exposed to lower HM contaminant. In such cases, continued HM accumulation can occur, leading to positive relationships between fish size and metal concentrations in tissues^1^.

In heavily polluted waters, the HM content of fish tissues may be higher by several folds than the permissible levels^45^. Therefore, consuming polluted fish at high rates can persuade many health problems for humans, particularly for most coastal residents who consider fish as the main dietary source of animal protein^46^.

### Risk assessment associated with consuming different sizes of *M. cephalus*

The present findings of risk assessment indexes underscore the importance of monitoring HM contamination in fish, particularly for populations that consume fish frequently^47^. Our data suggests a clear size-related pattern in HM accumulation and associated health risks, with smaller fish generally posing higher risks due to their higher concentrations of HM. The non-carcinogenic risk in the present study was expressed through the THQ, which is a significant risk assessment input associated to HM-polluted food ingestion. Consequently, the documented THQ values were > 1.0 for each HM independently, which suggests that fish are harmless for both habitual and normal human ingestion patterns. Meanwhile the THQ studies each HM individually, the THQ cannot be considered as a direct assessment of hazard^34^.

Although the present study reported a negligible and/or low carcinogenic risk, we documented non-carcinogenic risks for habitual consumers of all *M. cephalus* sizes from northern east area of Lake Manzala. While individual THQs were < 1, habitual consumers faced cumulative risks (HI > 1), warranting advisories for frequent consumption of small fish. Highlighting the potential health impacts of long-term exposure to HM contaminants through diet.

### Occurrence of Myxobolus parasite and histopathological lesions in *M. cephalus*

Myxobolus is a genus of myxosporean parasites that can infect various fish species, including *M. cephalus*. Fish parasites can produce proteolytic enzymes responsible for tissue deterioration and potentially parasite encystation. The activation of melanomacrophage centers plays a crucial role in developing an immune response to the parasite, facilitating the deposition of resistant pathogens like parasitic spores and antigen processing in immune responses. Water pollution, especially with HM, can severely impact aquatic ecosystems and the organisms within them. Heavy metals such as Pb, Hg, Cd, among others, are known to be toxic to aquatic organisms, including fish^48^.

Fish under stressful conditions require additional energy sourced from stored nutrients like proteins, fats, and carbohydrates to adapt. Certain metals (e.g., As, Cd, Cr, Cu, Fe, Hg, Ni, Pb, Zn) can generate reactive oxygen species (ROS) due to their redox potential, which play a role in maintaining fish physiology. However, an excess of ROS can lead to oxidative stress, damaging proteins, lipids, DNA, and affecting cellular function. These pollutants enter the marine environment through industrial discharges, agricultural runoff, and other human activities^49^. Anuprasanna et al.^50^ suggested that scientific literature supports the use of fish parasites as indicators of environmental contamination, indicating a connection between fish parasites and various contaminants, including toxic metals and sewage pollutants. Moreover, the statement proposes that fish parasites can aid their hosts in surviving in heavily metal-polluted environments. Beyond HMs, parasitic infections like *Myxobolus spp*. further compromise fish health, with potential synergies from HM-induced immunosuppression. Radwan et al.^51^ implied that this assertion is backed by scientific research, suggesting that fish parasites may accumulate larger quantities of heavy metals, acting as “metal sinks”. While larger fish hosted more parasites^52^, their lower HM levels suggest parasites may sequester metals^53^, though this requires further isotopic tracing to confirm. Székely and Molnár^54^ reported similar findings in the gills of sea bream infected with four Myxobolus species (*M. macrocapsularis*,* M. impressus*,* M. bramae*,* and M. hungaricus*), infecting the capillary network of the gill lamellae and respiratory plate. Dias et al.^55^ stated alterations in the capillary network, hyperplasia of gill epithelium, and structural disorganization of secondary lamellae in fish infested by parasite. Moreover, Chavda et al.^56^ reported hemorrhagic and necrotic changes in epithelia and connective tissues of the gills of major aquaculture carp (*Catla catla*) infested by myxozoan parasites.

Furthermore, fish exhibited severe congestion of submucosal blood vessels and perivascular hemorrhage. The histopathological analysis of gills infected with *Myxosporean sp*. revealed the accumulation of fibrinous exudates mixed with leukocytes and myxospores^45^. Similar hepatic alterations were recorded by Novoa et al.^57^ who stated inflammations, erythrocytes’ accumulation outside the blood vessels forming a red lump of blood, and fibrosis progressing to cirrhosis represents an irreversible process accompanied by subsequent mechanical effects, and neoplasia implications. MacKenzie et al.^58^ and Asad et al.^59^ postulated that this inflammatory response is to support the recovery process of tissue and suppress the causative agent of necrosis.

Moreover, parasite infections can lead to various cell alterations, including hepatic coccidiosis, which is characterized by the enlargement of oocysts and the formation of granulomas from connective tissue or fibrotic capsules, partially replacing the host liver parenchyma^60^, similar findings were observed in our results. Similarly, fish exposed to HM recorded hepatic lesions in the form of hemorrhage, necrosis, hepatocyte vacuolization, nuclear degeneration, and presence of macrophages^61^. Fatima and Usamani^8^ also reported hepatic histopathological alterations including vacuolization, nuclear pyknosis, blood vessel rupture, and bleeding in fish exposed to HM.

The present kidney histopathological findings are consistent with previous studies^62–64^, which demonstrated that asynchronous spore development leads to the compression and degeneration of tubular cells, resulting in changes in lumen size. Furthermore, the degree of pathogenicity can vary, with additional observations such as hyaline degeneration, glomerular congestion, cellular edema, and the formation of a connective capsule around the spores, as seen in kidney infections in the pacu species *P. mesopotamicus*^65^. Additionally, Oreochromis niloticus infected with *myxosporidian* parasites exhibited degenerative and necrotic changes in the lining epithelium of renal tubules, along with numerous extra-sporogenic and sporogenic stages of *myxosporidian* parasites, associated with the activation of melanomacrophage^66^.

The degenerative changes in the liver and kidneys of fish can lead to hypoxia, osmoregulatory failure, and death of the host. The degree of pathogenicity is influenced by various factors, including the *Myxosporean* species, its life cycle, biology, host species, age, nutritional status, and host resistance^21^. Pathogenicity can result in the rupture of cysts, leading to hemorrhages, blood loss, and granulomas^67^. In the present study, the liver exhibited a higher degree of pathogenicity due to the presence of spores: severe focal areas of congestion in small fish, focal areas of granulomas in medium-sized fish, and dilated veins with congestion surrounded by hyaline substances in large fish. Similarly, in kidney sections, medium-sized *M. cephalus* showed spores alongside large cysts containing numerous *myxobolus* at different stages, leading to a higher degree of pathogenicity compared to small and large sizes, which only showed a large number of spores.

Salaah and El-Gaar^68^ indicated that exposure to HM is related to negative health effects that can suppress immunity in fish, making them more susceptible to infections and parasitic diseases. *Myxobolus* infections have been reported in various fish species, with their prevalence and severity potentially influenced by environmental factors, including water quality and pollutants^51^. On the other hand, Mahmoud et al.^69^ concluded that while the precise mechanisms linking HM pollution and *Myxobolus* infection in *M. cephalus* fish may vary, several potential pathways exist. HM can induce physiological stress and compromise the fish’s immune system, thereby increasing susceptibility to infections. Moreover, HM might directly influence the life cycle and reproductive capabilities of *Myxobolus* parasites, potentially increasing infection rates in fish populations^70^. Based on the finding of Radwan et al.^51^, parasites might actually aid their hosts in surviving by sequestering these harmful metals in HM-polluted environments.

According to Bagge et al.^52^, the larger the fish, the more parasites they commonly harbor, while small host individual’s incapable of supporting relatively high parasite abundances^71,72^. Barber^73^, the most accurate predictor of parasite mass was the host’s growth rate, adjusted for body size. This suggests that the larger fish tended to develop the largest parasites. Therefore, larger-growing hosts seem to provide more favorable environments for parasite growth.

Moreover, Brázová^53^ there is a notable relationship between HM accumulation and parasitic infections in fish. Their research found that parasites can accumulate higher levels of HM compared to their host fish. Our findings align with these findings, as we recorded lower HM accumulation in medium - and large -sized fish compared to small-sized fish, as well as a higher presence of parasites in larger fish. This relationship suggests that while larger fish show lower levels of HM accumulation and health risks, they recorded higher parasitic infections. Such findings highlight the complex interactions between HM-parasite infection exposure in fish. Our findings directly inform SDG 2 (Zero Hunger) and 3 (Good Health) by identifying size-based consumption risks in a key Egyptian fishery.

## Conclusion

Our study examined heavy metal accumulation and Myxobolus parasite infections in *M. cephalus* from the northeastern part of Lake Manzala in context of fish size. Which is directly connected to SDG 2 (Zero Hunger) and 3 (Good Health) by identifying size-based consumption risks in a key Egyptian fishery. Reporting that smaller fish had higher levels of heavy metals and health risks compared to medium- and large-sized fish. Additionally, larger fish showed a higher prevalence of Myxobolus parasites. The histopathological analysis identified significant pathological changes in infected fish, including severe congestion, perivascular hemorrhage, and granuloma formation in the liver, kidneys, and gills. Our findings highlight the importance of developing and implementing guidelines and advisories for fish consumption, particularly for habitual consumers, as well as continuous monitoring and assessment of heavy metals and Myxobolus infections to protect fish health, ensure the sustainability of fish stocks, and safeguard human health. Future research should focus on the mechanisms linking heavy metal pollution and parasite infections, and on improving biosecurity measures in aquaculture to mitigate these risks.

## Data Availability

The datasets used and/or analysed during the current study available from the corresponding author on reasonable request.
